# Effects of Solution Chemistry and Aging Time on Prion Protein Adsorption and Replication of Soil-Bound Prions

**DOI:** 10.1371/journal.pone.0018752

**Published:** 2011-04-19

**Authors:** Samuel E. Saunders, Qi Yuan, Jason C. Bartz, Shannon Bartelt-Hunt

**Affiliations:** 1 Department of Civil Engineering, University of Nebraska-Lincoln, Peter Kiewit Institute, Omaha, Nebraska, United States of America; 2 Department of Medical Microbiology and Immunology, Creighton University, Omaha, Nebraska, United States of America; Ohio State University, United States of America

## Abstract

Prion interactions with soil may play an important role in the transmission of chronic wasting disease (CWD) and scrapie. Prions are known to bind to a wide range of soil surfaces, but the effects of adsorption solution chemistry and long-term soil binding on prion fate and transmission risk are unknown. We investigated HY TME prion protein (PrP^Sc^) adsorption to soil minerals in aqueous solutions of phosphate buffered saline (PBS), sodium chloride, calcium chloride, and deionized water using western blotting. The replication efficiency of bound prions following adsorption in these solutions was also evaluated by protein misfolding cyclic amplification (PMCA). Aging studies investigated PrP^Sc^ desorption and replication efficiency up to one year following adsorption in PBS or DI water. Results indicate that adsorption solution chemistry can affect subsequent prion replication or desorption ability, especially after incubation periods of 30 d or longer. Observed effects were minor over the short-term (7 d or less). Results of long-term aging experiments demonstrate that unbound prions or prions bound to a diverse range of soil surfaces can readily replicate after one year. Our results suggest that while prion-soil interactions can vary with solution chemistry, prions bound to soil could remain a risk for transmitting prion diseases after months in the environment.

## Introduction

Prion diseases, or transmissible spongiform encephalopathies (TSEs), are fatal neurodegenerative diseases that include chronic wasting disease (CWD, deer, elk, and moose), bovine spongiform encephalopathy (BSE or ‘mad cow’ disease), scrapie (sheep and goats), and Creutzfeldt-Jakob disease (CJD, humans) [1]. Strong evidence demonstrates the infectious agent of prion diseases is PrP^Sc^, an abnormally-folded isoform of a normal cellular protein, PrP^c^ [2]–[4]. The biochemical properties of PrP^Sc^ are distinct from PrP^c^ and include strong resistance to proteolysis and inactivation, increased hydrophobicity, and a propensity for aggregation [1], [5]. Infectious CWD and scrapie prions are shed from living hosts and present in mortalities and can remain infectious after years in the environment [6], [7]. The environment can serve as a reservoir of prion infectivity and may facilitate a sustained incidence of CWD in free-ranging cervid populations and complicate efforts to eliminate scrapie and CWD in captive herds [6], [7]. Soil and other environmental surfaces may act as significant environmental reservoirs of prion infectivity [7]–[9].

Prions enter the environment in complex, competitive matrices, such as urine, feces, saliva, blood, and birthing matter, as well as tissue from mortalities [6], [7]. PrP adsorption to soil and soil minerals has been previously characterized and variance in PrP adsorption with respect to prion strain and species, soil type, and PrP form (full length vs. N-terminally truncated) has been documented [7], [10]–[12]. Soil-bound prions are infectious [9], [13], but the mechanism(s) responsible for PrP adsorption to soil remain unknown. Various realistic scenarios may occur that lead to prion interactions with soil in a range of aqueous solutions, including highly concentrated biological solutions or relatively dilute surface or ground waters. Thus, solution chemistries with a range of pH values, ionic strengths, and ionic species are relevant to natural prion-soil interactions.

Dissolved ions present in solution can interact with both mineral surfaces and proteins to impact soil adsorption capacity and the conformation of adsorbed proteins. Prion adsorption studies to date have used a range of adsorption solutions including distilled/deionized (DI) water [12], [14]–[16], phosphate buffered saline (PBS or DPBS) [10], [11], [17]–[19], sodium acetate [20], [21], tris [21], [22], citric acid [22], sodium chloride (NaCl) [13], [19], [23], calcium chloride (CaCl_2_) [23], and 3-*N*-morpholinopropanesulfonic acid (MOPS) [13], [21], [22]. Only two studies have compared PrP adsorption as a function of solution chemistry, both using pure or enriched PrP sources. Ma et al. observed increased adsorption of enriched HY TME PrP^Sc^ with increasing ionic strength, reaching a plateau at 100 mM NaCl [21]. In another study, apparent differences in adsorption of recombinant PrP (recPrP, a model of PrP^c^) to montmorillonite were not observed using 150 mM NaCl or DI water, while adsorption was approximately 30% lower in 1X PBS [19]. Given the variation in the adsorption buffers used in previous prion studies, coupled with the use of pure PrP (recPrP or purified PrP^Sc^) in the absence of a competing organic matrix, such as tissue or excreta, it is unclear how solution chemistry affects PrP adsorption to soil from a brain homogenate matrix and whether such effects significantly impact prion replication efficiency and the risk of disease transmission via soil-bound prions.

The objectives of this study were to evaluate differences in prion adsorption and replication efficiency as a function of adsorption solution and to evaluate desorption and replication of soil-bound prions over time periods up to 1 year. We studied adsorption and desorption of HY TME hamster PrP^Sc^ to a range of soils and soil minerals for up to one year in various aqueous solutions. We also applied a previously developed semi-quantitative protein misfolding cyclic amplification (PMCA) protocol [9] to assay variance in the replication efficiency of PrP^Sc^ bound to soil minerals with respect to adsorption solution and aging time.

## Methods

### Prion Sources and PMCA substrates

Experiments were conducted using the hyper (HY) strain of transmissible mink encephalopathy (TME) infected hamster brain homogenate. Syrian hamsters were intracerebrally inoculated with the HY TME agent and sacrificed at terminal disease as described elsewhere [24]. Hamster brains were homogenized to 10% (w/v) in sterile Dulbecco's phosphate-buffered saline (DPBS) without Ca^2+^ or Mg^2+^ (Mediatech, Herndon, VA) or deionized (milli-Q) water using a Tenbroeck tissue grinder (Kontes, Vineland, NJ) dedicated to the HY TME strain. Clarified brain homogenate was prepared by collecting the supernatant of a 100×g, 5 min centrifugation. For the sand experiments, brain homogenate was digested with proteinase-K (30 min, 37°C, 25 g/ml, Roche Diagnostics Corporation, Indianapolis, IN). Digestion was stopped with pefabloc (100 µg/µl, Roche Diagnostics). For PMCA substrates, uninfected hamster brains were homogenized to 10% (w/v) in ice-cold conversion buffer (DPBS (pH 7.4) containing 5 mM EDTA, 1% (vol/vol) Triton X-100, and Complete protease inhibitor tablet (Roche Diagnostics)). Brain homogenates were then centrifuged at 500×*g* for 30 s and the supernatant was collected and stored at −80°C.

### Adsorption Solutions

This study used four adsorption solutions: DPBS without Ca^2+^ or Mg^2+^ (a model of biological fluids consisting of 137 mM NaCl, 2.7 mM potassium chloride (KCl), 10 mM sodium dibasic phosphate (Na_2_HPO_4_), and 2 mM potassium dibasic phosphate (KH_2_PO_4_)), 10 mM CaCl_2_ (a model for groundwater), 10 mM NaCl, or milli-Q (DI) water. The calculated ionic strengths of each of these solutions are 225 mM, 30 mM, 10 mM, and 0 mM, respectively. All solutions were either at neutral pH (NaCl, CaCl_2_, DI water) or 7.4 (DPBS).

### Prion Adsorption Assays

Gamma-irradiated fine white sand (Fisher Scientific, Pittsburgh, PA), Rinda silty clay loam soil (a Vertic Epiaqualf), sodium bentonite clay (CETCO, Arlington Heights, IL), silicon dioxide powder (Sigma Aldrich, St. Louis, MO), and humic acid-coated silica gel particles (SiO_2_-HA) [25] were used as sorbents and have been described previously [10], [26]. A 10% w/v clarified or crude brain homogenate was mixed with each soil in either 1X DPBS, DI water, 10 mM CaCl_2_, or 10 mM NaCl. The mixtures were rotated at 24 rpm (Mini Labroller, Labnet, Edison, NJ) at 22°C. Brain homogenate controls without soil were prepared in the same manner. Soil and brain homogenate concentrations and incubation times were selected based on previously published results [10], [11], [26] and are detailed in Table S1. After incubation, soil-BH mixtures were centrifuged at 100×g for 5 min. The supernatant was removed and fresh solution was added. The resuspended mixture was again centrifuged and the supernatant (‘first wash’) was collected. This was repeated once more to obtain the final soil pellet. Washing solutions were the same as the adsorption solution for each sample. The original supernatant, first wash, and pellets were collected and stored at −80°C. We have previously demonstrated that subsequent washes do not contain measurable PrP [10]. However, to assure that unbound PrP^Sc^ was not present in PMCA reactions, sand and SiO_2_-HA samples were washed five times prior to PMCA.

### Soil-Bound PrP Aging Assays

HY TME was equilibrated with silty clay loam soil, bentonite, SiO_2_ powder, fine quartz sand, and SiO_2_-HA using the parameters shown in Table S1. Following centrifugation and washing, soils were resuspended in a minimal amount of 1X DPBS or DI water and incubated undisturbed in sealed polypropylene tubes for up to 365 d at room temperature. Samples were collected at prescribed time points for each soil and resuspended to the desired concentration. A 10% brain homogenate was incubated as an unbound control. Gamma irradiated soils and sterile DPBS or DI water were used.

### PMCA

Protein misfolding cyclic amplification (PMCA) was performed as described previously [9]. Briefly, sonication was performed with a Misonix 4000 sonicator (Farmingdale, NY) with output set to 75V, generating an average output of 160 W during each sonication cycle. Before each PMCA round, an aliquot was placed at −80°C as an unsonicated control. After the first round of PMCA, an aliquot of the sonicated sample was added to fresh 10% (w/v) uninfected brain homogenate in conversion buffer and subjected to a second round of PMCA. The initial ratio of sample to uninfected brain homogenate was 1∶100 (see Table S1 for soil amounts loaded), and one round consisted of 144 cycles of 25 seconds of sonication followed by 10 minutes of incubation at 37°C. Homogenates from Round 1 were diluted 1∶1 for Round 2. Samples containing only uninfected brain homogenate were run with each round of PMCA as negative controls. None of these samples ever yielded detectable amounts of PrP^Sc^.

### Immunoblot analysis

A 96-well immunoblot assay as described previously [27] was used without modification to quantify unbound PrP in the supernatants and washes. SDS-PAGE and western blotting were used to detect bound PrP as described previously [24], [26]. Proteinase-K (PK) digestion was carried out as above, and digestion was terminated by boiling in SDS-PAGE sample buffer. The volumes of soil sample loaded into each well are shown in Table S1. All controls were 2.0 or 2.5 µl 10% HY TME BH. Blots were probed with mAb 3F4 (Millipore, Billerica, MA, 1∶10,000 dilution), which reacts with residues 110–113 (MKHM) of hamster PrP. Western blots were developed with Pierce Supersignal West Femto maximum-sensitivity substrate and imaged on a Kodak 2000R imaging station (Kodak, Rochester, NY). None of the soils used exhibit nonspecific binding to either the primary or secondary antibody [26]. Blot images were analyzed as described previously [26]. Net intensities of sample replicates (n =  3) were normalized as a percentage of the average of control HY BH replicates (n = 4) run on the same gel. Statistical analysis (t-tests assuming unequal variances) was performed using GraphPad Prism.

## Results

### Short-Term Effects of Adsorption Solution on PrP Adsorption and Desorption

**Figure 1:**
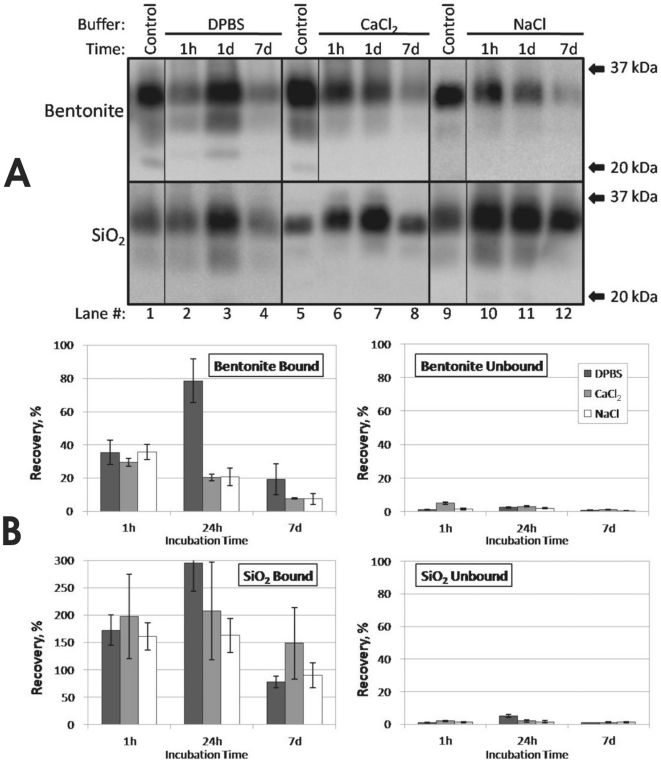
Solution Chemistry Does Not Affect Short-Term HY TME PrP^Sc^ Adsorption to Bentonite Clay and Silicon Dioxide Powder. (**A**): Representative Western blots of PrP^Sc^ adsorbed to bentonite clay or silicon dioxide powder (SiO_2_) in DPBS, CalCl_2_, or NaCl solutions for 1 h, 1 d, or 7 d at 22°C. ‘Control’ indicates 250 µg eq clarified HY TME brain control (DPBS for lane 1, H_2_O for lanes 5 and 9). (**B**): Approximate PrP^Sc^ recovery in soil pellets (‘Bound’) and supernatants (‘Unbound’). Error bars represent ±1 standard error of the mean.

We evaluated HY TME PrP^Sc^ adsorption to silicon dioxide (SiO_2_) powder and bentonite clay through 7 d in DPBS, CaCl_2_, and NaCl (Figure 1). In these experiments, brain homogenate was clarified prior to mixing with soil by centrifugation at 100× g for 5 min. Results using crude brain homogenate had similar trends with respect to adsorption solution, although recovery of adsorbed PrP^Sc^ was lower for both bentonite and SiO_2_ samples (data not shown).

For adsorption experiments conducted with bentonite and SiO_2_, less than 10% of total PrP^Sc^ was detected in all supernatants after 1 h (Figure 1), indicating over 90% of the PrP^Sc^ was adsorbed. This suggests minimal differences in the amount of PrP^Sc^ that sorbs as a function of adsorption solution. Recovery of PrP^Sc^ bound to bentonite (which requires desorption) was approximately 35% for all three solutions after 1 h (Figure 1). Recoveries decreased slightly for CaCl_2_ and NaCl at 1 d and 7 d, but increased to near 80% for DPBS at 1 d. Analogous increases were seen in unbound DPBS controls and in SiO_2_ DPBS samples (Figures S1 and 1B), suggesting an enhanced ability to detect PrP^Sc^ in DPBS after 1 d incubation. Recoveries of PrP^Sc^ bound to SiO_2_ were greater than or equal to 80% for all three solutions at all time points (Figure 1). The highest signal intensity obtained (300% higher relative to controls) was for DPBS at a 1 d incubation period (Figure 1A lane 3 and Figure 1B). It is possible that interaction with SiO_2_ greatly increased PrP^Sc^ detection, resulting in higher signal intensities. A high level of variance was seen in SiO_2_ sample replicates, especially for experiments conducted in CaCl_2_.

A significant difference (α = 0.05) in PrP^Sc^ desorption from bentonite was not observed as a function of adsorption solution except at 1 d. Significant difference in desorption from SiO_2_ was not observed between solutions at any of the three time points. PrP^Sc^ bentonite adsorption experiments using a 4-fold higher dose of clarified brain homogenate yielded similar results except a larger amount of PrP^Sc^ remained unbound in solution (data not shown). Differences in glycosylation patterns or migration of PrP^Sc^ was not observed between adsorption solutions for either mineral (Figure 1A).

To control for variances in tube adsorption, PrP degradation, and PrP detection, an unbound control was examined for each solution at the same dilution used for the adsorption experiments. One h and 1 d recovery of PrP was between 70–120% for DPBS and NaCl solutions but only 25–35% for CalCl_2_ (Figure S1). It is unclear why recovery of unbound PrP^Sc^ in CaCl_2_ was lower. Brain homogenate components visibly coagulate in CaCl_2_, which could inhibit PrP^Sc^ detection using the 96-well immunoassay. Alternately, PrP^Sc^ tube adsorption may have been higher in the CalCl_2_ solution, although a similar trend was not seen with bentonite or SiO_2_ sample recoveries in CaCl_2_ solution (Figure 1B). Differences in PrP^Sc^ abundance were not observed using either western blot or 96-well immunoblot between 0 hr HY TME controls homogenized in pure H_2_O or DPBS (data not shown), indicating that there is no inherent difference in PrP detection ability between these homogenate solutions. Recovery for all three solutions was lower at 7 d (15–35%, Figure S1), consistent with previous results indicating decreases in HY TME PrP in brain homogenate incubated at room temperature [28].

### Effect of Adsorption Solution on Soil Mineral-Bound Prion Replication

We investigated variance in the ability of soil mineral-bound prions to replicate (i.e. convert PrP^c^ to PrP^Sc^) when different adsorption solutions were used. We have previously used protein misfolding cyclic amplification (PMCA) to quantitatively compare the replication efficiencies of soil-bound prions [9]. There is a marked relationship between PMCA amplified signal and HY TME infectious titer [9]. For PMCA studies, we used a PrP to solids ratio one-half that used in the adsorption studies presented above to ensure no unbound PrP^Sc^ was present (Table S1). Uninfected negative PMCA controls did not yield a detectable PrP^Sc^ signal (data not shown), and previous work demonstrates the presence of soil particles does not lead to spontaneous PrP^Sc^ formation through at least three serial PMCA rounds [9].

**Figure 2:**
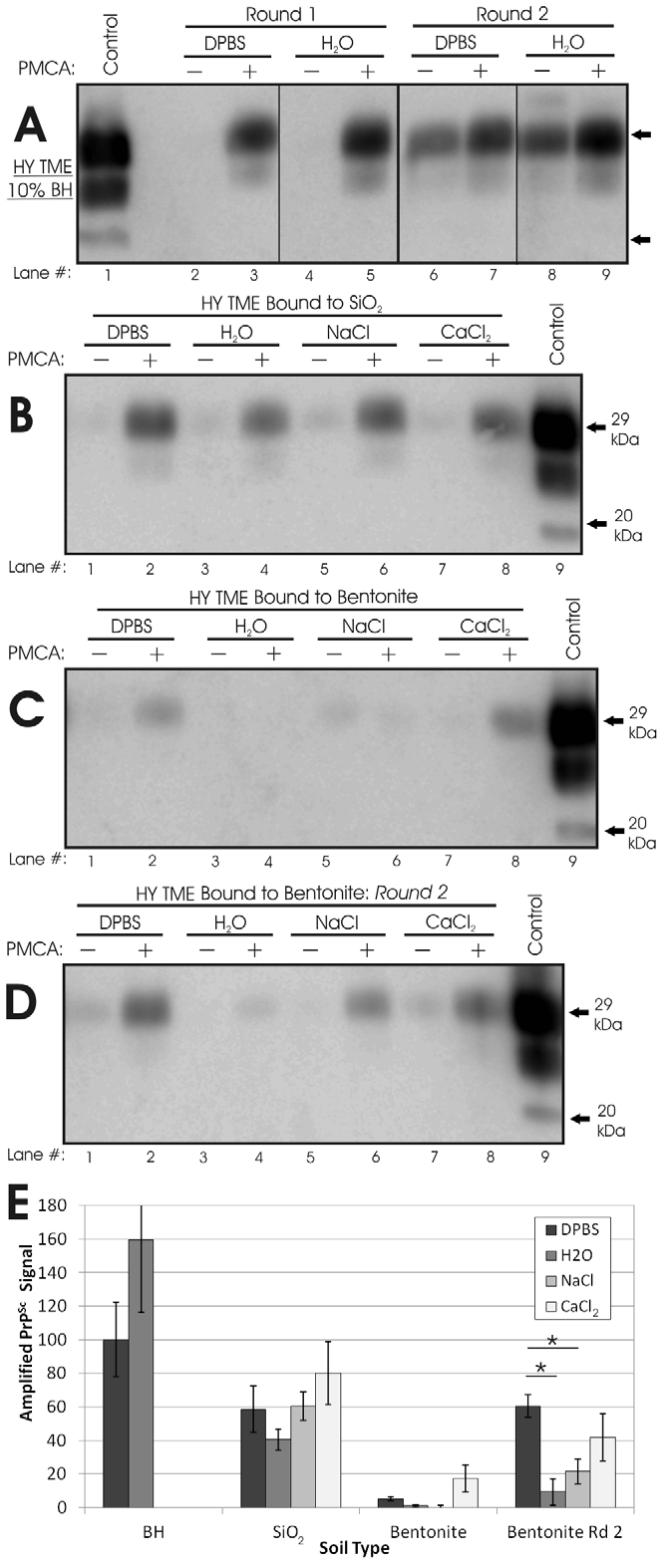
Effect of Adsorption Solution on Replication Efficiency of Soil Mineral-Bound HY TME. (**A**–**D**): Representative Western blots of HY samples subjected or not subjected to PMCA, shown with a 2 µl 10% BH control. (**A**): HY brain tissue homogenized in DPBS or H_2_O. (**B**): SiO_2_ powder-bound HY (one round PMCA). (**C**): First round of bentonite clay-bound HY. (**D**): Second round of bentonite clay-bound HY. (**E**): Quantification of blots shown in (A–D). Amplified signal was calculated by normalizing sample intensities to HY DPBS BH controls subjected to PMCA concurrently. Error bars show ±1 standard error of the mean. *Denotes significant difference (p<0.01).

We first assessed whether adsorption solution affects replication efficiency of unbound HY TME. Significant difference in the first or second round amplified signals of HY TME brain tissue homogenized in DI water or 1X DPBS were not observed (Figure 2A and 2E). We have previously shown that the presence of soil particles inhibits PMCA of unbound HY TME by 40–50%, but that this inhibition is consistent across soil/mineral types [9]. To evaluate whether the adsorption solution alters soil particle inhibition of HY PrP^Sc^ PMCA replication, we spiked HY TME into a solution of SiO_2_ or bentonite suspended in both DPBS and H_2_O and did not identify significant (α = 0.05) differences in inhibition levels (data not shown). Therefore, adsorption solution does not alter the PMCA efficiency of unbound HY TME or HY TME in the presence of soil particles.

Only minor differences in PMCA efficiency were observed between HY TME bound to bentonite and SiO_2_ in DPBS, NaCl, CaCl_2_, and DI water (Figure 2). HY TME bound to SiO_2_ in DI water had the lowest amplified first round signal (40% compared to the unbound control), while DPBS (59%), NaCl (60%), and CaCl_2_ (80%) were higher (Figure 2B and 2E). However, none of these differences were significant (p>0.10). All bentonite samples had low levels of amplification (0–17%) after one round (Figure 2C and 2E), agreeing with previously reported results indicating a significant decrease in HY replication ability upon binding to bentonite in DPBS solution [9]. Larger differences between bentonite samples were observed after a second PMCA round, where DPBS samples had a significantly higher amplified signal (60%) than H_2_O (10%) and NaCl (20%) (p< 0.01)(Figure 2D and 2E). Bentonite in CaCl_2_ (42%) also amplified more than bentonite in H_2_O or NaCl, but was not significantly different than the other solutions tested (α = 0.05). In contrast to bentonite and SiO_2_, adsorption solution appeared to have a profound effect on HY TME bound to humic acid (HA-coated silica gel particles, SiO_2_-HA). HY TME bound to SiO_2_-HA in DPBS readily amplified PrP^Sc^ whereas DI water samples did not amplify PrP^Sc^ through two PMCA rounds (Figure S2).

It was noted that SiO_2_ particles in H_2_O did not pellet as readily as in other solutions and some particles were visibly present in the supernatant. Thus, supernatants of each SiO_2_ and bentonite sample were also subjected to PMCA to determine if unpelleted soil-bound PrP^Sc^ contained significant replication ability. For bentonite, all supernatants yielded blank or extremely faint amplified signals (0–5%) after one round (data not shown). PrP^Sc^ was not detected after one round in supernatants obtained from solutions of SiO_2_ powder in DPBS, NaCl, and CaCl_2_ (data not shown). In contrast, the amplified PrP^Sc^ signal from the supernatant of SiO_2_ in DI water was 30%, consistent with the observation of visible particles in the supernatant and lower amplified signal of the corresponding pellets (Figure 2C).

**Figure 3:**
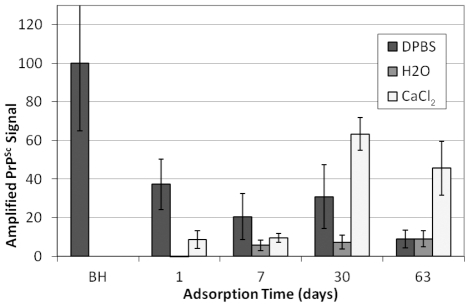
Effect of Adsorption Time and Adsorption Solution on Sand-Bound HY TME Replication. Amplified PrP^Sc^ signals from one round of PMCA of HY TME sand samples. Amplified PrP^Sc^ signals were normalized to HY DPBS BH controls subjected to PMCA concurrently. Error bars show ±1 standard error of the mean. Representative Western blots shown in Figure S3.

Replication of prions bound to sand was also investigated as a function of adsorption solution and time (Figure 3). Previous results indicate that maximum HY adsorption to sand in DPBS occurs at a BH:soil ratio of approximately 150 µg brain/mg sand [11] and can require up to 30 d [10]. Moreover, proteinase-K (PK) digestion of the brain homogenate prior to mixing with sand yields much higher PrP adsorption [11]. Thus, for the present experiments, HY BH was digested with PK prior to incubation with sand (Table S1). Sand-BH incubation times of 1–63 d yielded similar PMCA efficiencies for DPBS (Figures 3 and S3A). Amplified signals ranged from 9–38% but were not significantly different (α = 0.05). DI water samples yielded lower PMCA amplification, with a failure to detect PrP^Sc^ from samples with 1 d incubations and amplified PrP^Sc^ signals of 6–9% for 7–63 d incubations (Figures 3 and S3B). Amplification from CaCl_2_ was 9% for 1 and 7 d incubations and increased to 63 and 46% at 30 and 63 days, respectively (Figures 3 and S3C). Thus, adsorption time affected replication efficiency for sand in CaCl_2_ and DI water but not DPBS.

### Long-Term Incubation of Unbound and Soil-Bound PrP^Sc^

**Figure 4:**
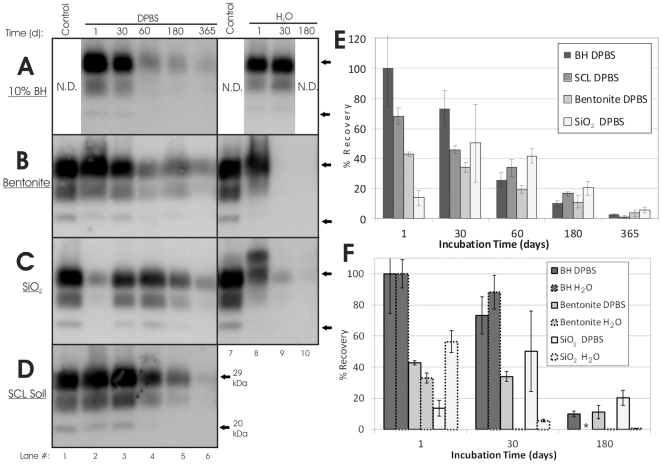
Long-Term Aging of Unbound and Soil-Bound HY TME PrP^Sc^. (**A**–**D**): Representative Western blots (n = 3) of aged HY TME samples, shown with a 2.5 µl 10% BH control. Arrows indicate migration of 29 kDa and 20 kDa molecular weight markers. (**A**): 10% HY brain homogenate. (**B**): Bentonite clay. (**C**): Silicon dioxide powder. (**D**): Rinda silty clay loam soil (SCL Soil). (**E**–**F**): Quantification of blots shown in (A–D). Error bars show ±1 standard error of the mean. *indicates sample (BH H_2_O 180 d) not available.

To evaluate soil-bound PrP^Sc^ fate upon long-term incubation, HY TME was adsorbed to sterile soil (Table S1) and then incubated undisturbed in sealed tubes at room temperature for up to one year. Trends in recovery of PrP^Sc^ from bentonite and silty clay loam soil (SCL soil) aged incubated in DPBS were similar to unbound BH controls (Figure 4A, 4B, 4D, and 4E). One day recoveries of PrP^Sc^ bound to bentonite and SCL soil were 43% and 68% respectively, and decreased to 4% and 1%, respectively at 365 d (Figure 4B and 4D). Unbound PrP^Sc^ in DPBS also decreased through the incubation period from 100% at 1 d to 3% at 365 d (Figure 4A and 4E). Recovery of PrP^Sc^ from SiO_2_ powder DPBS samples was variable, with 14% recovery after 1 d, 50% recovery for 30 d, and 6% recovery at 365 d (Figure 4C and 4E). PrP^Sc^ bound to sand and SiO_2_-HA was not detectable at 30 d (data not shown), consistent with previous results showing loss of WB detection after 7 d [26]. This may be due in part to lower levels of initial bound PrP^Sc^.

Differences in recovery rates were not observed between soil samples digested or not digested with proteinase-K (PK) prior to SDS-PAGE detection, although recovery of unbound PrP was increased with PK digestion (data not shown). N-terminal truncation of PrP, denoted by a clear shift in immunoblot migration, was observed for both bound and unbound samples aged 30 d or longer when PK-digestion was not used (data not shown), which is consistent with previous results [28].

Contrasting results were found for aged PrP^Sc^ bound to bentonite and SiO_2_ in DPBS and DI water. Whereas PrP^Sc^ was readily detected after one year in solutions of bentonite and SiO_2_ in DPBS (Figures 4B and 4C, lane 6), PrP^Sc^ was not detected at 30 d (bentonite) and 180 d (SiO_2_) for DI water samples (Figures 4B, 4C, and 4F). In contrast, the unbound PrP^Sc^ signal from samples incubated in DI water remained strong after 30 d (Figure 4A, lane 9), the longest time point examined. PK-digestion was not effective at truncating PrP^Sc^ bound to SiO_2_ powder after 1 d (Figure 4C, Lane 8), although the average recovery was 56% for the 1 d samples incubated in DI water compared to 14% for the samples incubated in DPBS.

### Replication of Aged Soil-Bound Prions

**Figure 5:**
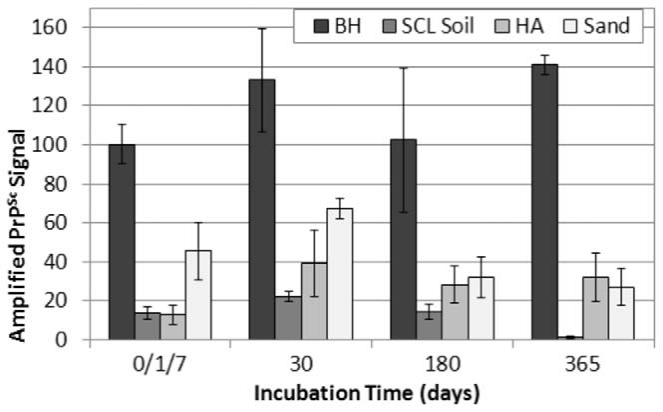
Soil-Bound HY TME Prions Remain Replication Competent after 1 Year Incubation. Amplified PrP^Sc^ signals from one round of PMCA of HY TME unbound or bound to various soils. Amplified PrP^Sc^ signals were normalized to HY DPBS BH controls subjected to PMCA concurrently. Error bars show ±1 standard error of the mean. Representative Western blots shown in Figure S4.

Aged samples were subjected to one round of PMCA to determine if changes in the ability of unbound and soil-bound PrP^Sc^ to replicate occurred over time. The average PMCA efficiency of unbound prions did not vary significantly (p>0.05) from 0 to 180 d, while the 365 d samples exhibited an increase of 40% compared to 0 d controls (Figures 5 and S4A). This is in contrast to the PrP^Sc^ western blot results, which showed steady declines in PrP^Sc^ abundance through 365 d (Figure 4A). PMCA replication efficiency of HY TME bound to SCL soil was also constant through 180 d, but near zero amplification was seen in the 365 d samples (Figure 5 and S4B lane 9). These results are more consistent with the western blot results, which showed very low or undetectable amounts of PrP^Sc^ bound to SCL soil after 365 d (Figure 4B, lane 6).

Aged bentonite and SiO_2_ samples were generated using a soil to PrP ratio that did not yield complete adsorption of PrP^Sc^ (data not shown). Given that the presence of unbound PrP^Sc^ could significantly alter PMCA results, PMCA was not performed on these samples. However, since sand and humic acid (SiO_2_-HA) samples could be washed to eliminate all detectable unbound PrP^Sc^ without reducing sample mass, these samples were used for PMCA analysis. Note that PrP adsorption for aged sand and SiO_2_-HA samples was conducted using 30 d and 14 d incubation times, respectively (Table S1), due to previous data indicating slow adsorption kinetics for these minerals [10], [26]. Although PrP^Sc^ bound to sand and SiO_2_-HA samples was undetectable for 30 d and beyond (data not shown), these samples readily amplified PrP^Sc^ (Figures 5, S4C, and S4D). Amplified PrP^Sc^ from both SiO_2_-HA and sand increased 20–25% from 1 and 7 d, respectively, to 30 d, consistent with previous results showing increasing adsorption of PrP to sand through 30 d [10]. Sand PMCA efficiency decreased from 30 d (67%) to 180 d (32%) but remained strong through 365 d (27%), as did the 365 d SiO_2_-HA samples (32%). In contrast, when DI water was used as the adsorption and incubation solution for SiO_2_-HA samples, PrP^Sc^ was not detected after one or two rounds of PMCA for samples incubated 1 to 410 d (Figure S2).

## Discussion

### Effects of Solution Chemistry on Prion Adsorption and Replication

A key objective of this study was to determine if adsorption solution chemistry significantly affects prion protein adsorption and soil-bound prion replication. Prions could exist in the environment in a range of aqueous solutions from high ionic strength biological solutions originating from excreta or an infected mortality to relatively dilute surface or ground waters. Important determinants of the solution chemistry of ‘natural’ prion-soil interactions could include soil and water composition as well as precipitation and other weather-related factors. Although previous studies evaluating prion adsorption to soil have used a wide range of adsorption solutions, only two studies have compared PrP adsorption as a function of solution chemistry, both using PrP sources in the absence of a biological matrix [19], [21].

In the present study, we used infectious brain homogenate to study PrP adsorption and desorption as well as soil-bound prion replication (i.e. conversion of PrP^c^ to PrP^Sc^). We found only slight differences in PrP^Sc^ adsorption to SiO_2_ powder and bentonite clay in DPBS, NaCl, and CaCl_2_ (Figure 1). Differences in SiO_2_- and bentonite-bound prion replication were also slight between solutions (Figure 2), although it should be noted that small differences in PMCA amplification can be indicative of much larger differences in infectious titer [9]. Moreover, adsorption solution significantly affected humic acid-bound prion replication, with SiO_2_-HA samples incubated in DI water showing complete inhibition of replication (Figure S2). Sand-bound prion replication also varied with adsorption solution, although all solutions generated sand-bound prions capable of replication (Figure 3). In general, use of DI water as the adsorption solution yielded lower soil-bound prion replication compared with DPBS and CaCl_2_ (Figures 2, 3, and S2). This trend was not mirrored in unbound prion controls (Figure 2A), indicating that soil adsorption was responsible for the differences in behavior between solutions. Long-term mineral-bound PrP^Sc^ fate was markedly different when using a DI water solution compared with DPBS (Figure 4). While PrP^Sc^ bound to SiO_2_ and bentonite in DPBS was readily detected after 365 d, it was faint or undetectable after 30 d in DI water. As with the short-term replication studies, these contrasting results were not observed in unbound controls, further demonstrating that soil adsorption can affect PrP^Sc^ replication.

The specific mechanisms of prion adsorption remain unknown, and thus interpretation of variance in PrP-soil interactions with different adsorption solutions is challenging. Studies investigating the effect of solution chemistry on protein adsorption, especially those using environmentally-relevant sorbents (soil minerals), are extremely limited, and in all such studies, protein-surface interactions were investigated using pure protein solutions. It is known that ions present in protein-soil solutions can interact with both mineral surfaces and proteins to influence surface adsorption capacity as well as the adsorbed protein conformation. For example, monovalent cations in solution can inhibit protein adsorption to mica [29], and phosphate can stabilize protein structure during adsorption [30], as well as retard adsorption and increase the adsorbed protein footprint [31]. In addition, phosphate has been shown to compete with negatively-charged proteins when binding to certain surfaces [32], but conversely, the binding of four proteins to montmorillonite and kaolinite clays increased in phosphate buffer compared to DI water [33]. Our results suggest that components in phosphate buffer (phosphate, sodium, potassium, or chloride ions) may stabilize PrP^Sc^ and/or alter PrP^Sc^-soil interactions such that soil-bound PrP^Sc^ is more readily available and able to convert PrP^c^ and thereby replicate. A calcium chloride solution also appears to exhibit a similar influence as DPBS on PrP^Sc^-soil interactions compared with DI water. A rigorous examination of the effect of solution ionic strength and ionic species on PrP interactions with soil in the presence of a competitive organic matrix would be required to determine the physiochemical mechanisms responsible for the observed differences between adsorption solutions.

The results of this study have significant implications for both previous and future studies of prion-soil interactions as well as prion fate in the environment. Previous PrP adsorption studies have used a wide range of adsorption solutions, which may hinder comparison and interpretation of results across studies. Given that solution chemistry has been shown to be an important variable in pure protein adsorption studies [19], [21], [29]–[33], caution is clearly warranted when interpreting or designing PrP-soil studies using pure recPrP or enriched PrP^Sc^. However, our results suggest that although solution chemistry is an important consideration for prion adsorption, it may be less significant in short-term studies that use brain homogenate or excreta as a prion source.

### Effects of Soil-Bound Aging on Prion Replication

The other key objective of this study was to evaluate long-term PrP-soil dynamics. The environment can serve as a long-term reservoir of CWD and scrapie infectivity [34], [35] and prions bound to soil retain infectivity [9], [13], [23], but it remains unclear whether soil-bound prions play a significant role in indirect CWD and/or scrapie transmission. The ability of soil-bound prions to retain infectivity over many months or years is unknown, and therefore the potential for prion disease transmission via contaminated soil cannot be accurately assessed. The results of the present study could be considered a conservative scenario for long-term prion survival in soil environments. Sterile soil and brain homogenates were used, and soils were kept saturated at room temperature and were not exposed to any potential environmental degradants such as soil microorganisms, heat, freezing, and drying [7]. Our results demonstrate that both unbound and soil-bound prions maintain high levels of replication efficiency after 1 year (Figure 5) and thus most likely do not lose significant levels of infectivity.

Although replication efficiency was conserved through 1 year, we did observe decreases in soil-bound and unbound PrP^Sc^ over time (Figure 4). Decreases in PrP^Sc^ detection over the aging period could be due to a number of factors. Unbound samples were subject to degradation due to natural proteases or chemicals present in brain homogenate. Soil-bound PrP samples were also subjected to natural proteases as well as degradants present in soil (e.g. oxidants) not affected by gamma-irradiation. Given that we observed decreases in the ability to detect PrP^Sc^ from both unbound and soil-bound aged samples but no such decreases in sample replication efficiency (except with SCL soil at 365 d, Figure S4B), the PrP^Sc^ population responsible for initial replication seeding may be small and not generally affected by aging. Variance in the ability to detect PrP^Sc^ over time may also account for this discrepancy, where aging processes render PrP^Sc^ undetectable but still capable of seeding PrP^c^ conversion.

The present results are generally consistent with previous studies of long-term unbound and soil-associated PrP survival. Detectable BSE and scrapie PrP^Sc^ from brain homogenate was shown to survive 140 d incubation at 20°C in PBS [36] and for 6 months at 16°C in water containing two mild detergents [12]. Detectable amounts of soil-bound BSE and scrapie PrP^Sc^ were observed following 18 months incubation at 16°C [12]. Additionally, the 263K agent remains infectious following burial of BH-soil mixtures for up to 3 years [18], [37]. It must be noted that the present study used HY TME hamster prions which, although used extensively for previous prion-soil experiments [9]–[11], [13], [21], [23], [26], are not naturally-occurring and may not accurately simulate CWD or scrapie fate in the environment [6], [7], [12], [28]. Moreover, our results do not consider prion infectivity, only PrP^Sc^ levels and replication efficiency. While PrP^Sc^ levels are not necessarily indicative of infectious titer [38], [39], our previous studies using PMCA indicate a strong correlation between PMCA replication efficiency and infectious titer [9].

Following prion entry into the soil environment, the heterogeneity in the prion source (i.e. an organic matrix such as tissue or excreta) combined with soil heterogeneity results in an extremely complex system for prion-soil interactions [7]. The characteristics of prion interactions with soil, including the soil type [9], [10], [12], [26], prion strain and species [11], [12], [28], soil:PrP ratio [11], kinetics of prion-soil interactions [9]–[11], [28], and solution chemistry, can have significant effects on prion adsorption and subsequent fate and transmission. Overall, the influence of solution chemistry on prion desorption and replication was slight in this study, especially in short term experiments (≤7 d), and our results indicate that prions bound to soil in contact with a range of solution chemistries could remain a risk for transmitting prion diseases after long periods in the environment.

## Supporting Information

Figure S1 Unbound PrP^Sc^ adsorption solution controls.
(DOC)

Figure S2 Replication of humic acid (SiO_2_-HA)-bound HY TME.
(DOC)

Figure S3 Representative immunoblots of sand-bound PMCA.
(DOC)

Figure S4 Representative immunoblots of aged soil-bound PMCA.
(DOC)

Table S1 Parameters of PrP adsorption to soil and soil minerals.
(DOC)
