# Supplementary material for: Effects of Solution Chemistry and Aging Time on Prion Protein Adsorption and Replication of Soil-Bound Prions
Source: PLoS One. 2011 Apr 19;6(4):e18752. doi: 10.1371/journal.pone.0018752 (PMC3079715; doi:10.1371/journal.pone.0018752)
Supplement: Figure S1 — Unbound PrPSc adsorption solution controls. (DOC) [file pone.0018752.s001.doc]

*
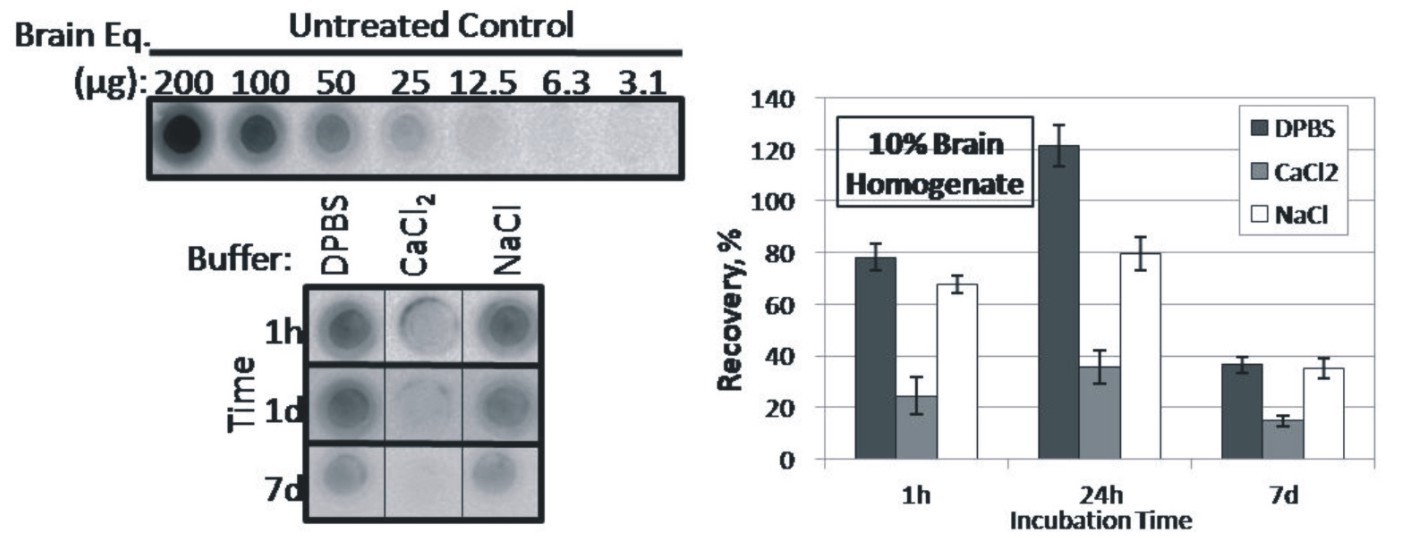
*

**Figure S1**. Unbound PrP^Sc^ adsorption solution controls. At left, representative (n=3) 96-well immunoblots of incubated controls in 1X DPBS, 10 mM CalCl_2_, or 10 mM NaCl. A HY TME 10% BH (clarified) standard curve is also shown. All blots used mAb 3F4 and were digested with PK. At right: calculated recovery of controls. Blots were quantified using the standard curve as in Saunders et al. [1].

**Literature Cited**

1. Saunders SE, Bartz JC, Bartelt-Hunt SL (2009) Prion protein adsorption to soil in a competitive matrix is slow and reduced. Environmental Science & Technology 43: 7728–7733.
