# Supplementary material for: Effects of Solution Chemistry and Aging Time on Prion Protein Adsorption and Replication of Soil-Bound Prions
Source: PLoS One. 2011 Apr 19;6(4):e18752. doi: 10.1371/journal.pone.0018752 (PMC3079715; doi:10.1371/journal.pone.0018752)
Supplement: Figure S2 — Replication of humic acid (SiO2-HA)-bound HY TME. (DOC) [file pone.0018752.s002.doc]

**
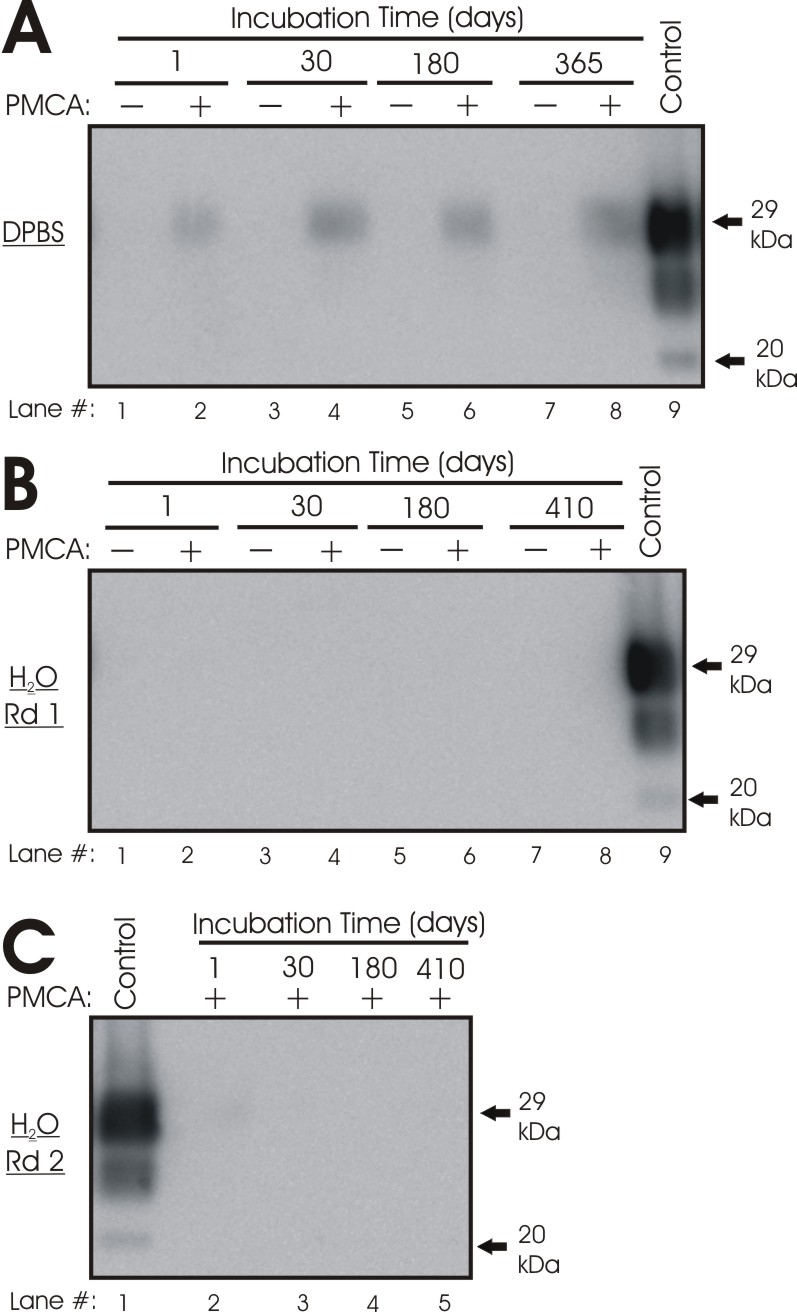
**

**Figure S2**. Humic acid (SiO_2_-HA)-bound HY TME PMCA. **(A-C):** Representative blots of HY SiO_2_-HA samples subjected or not subjected to PMCA, shown with a 2 µl 10% BH control. All samples PK-digested and blotted with mAb 3F4. **(A):** HY SiO_2_-HA DPBS first PMCA round. **(B):** HY SiO_2_-HA DI water first PMCA round. **(C):** HY SiO_2_-HA DI water second PMCA round.
