# Supplementary material for: Effects of Solution Chemistry and Aging Time on Prion Protein Adsorption and Replication of Soil-Bound Prions
Source: PLoS One. 2011 Apr 19;6(4):e18752. doi: 10.1371/journal.pone.0018752 (PMC3079715; doi:10.1371/journal.pone.0018752)
Supplement: Figure S3 — Representative immunoblots of sand-bound PMCA. (DOC) [file pone.0018752.s003.doc]

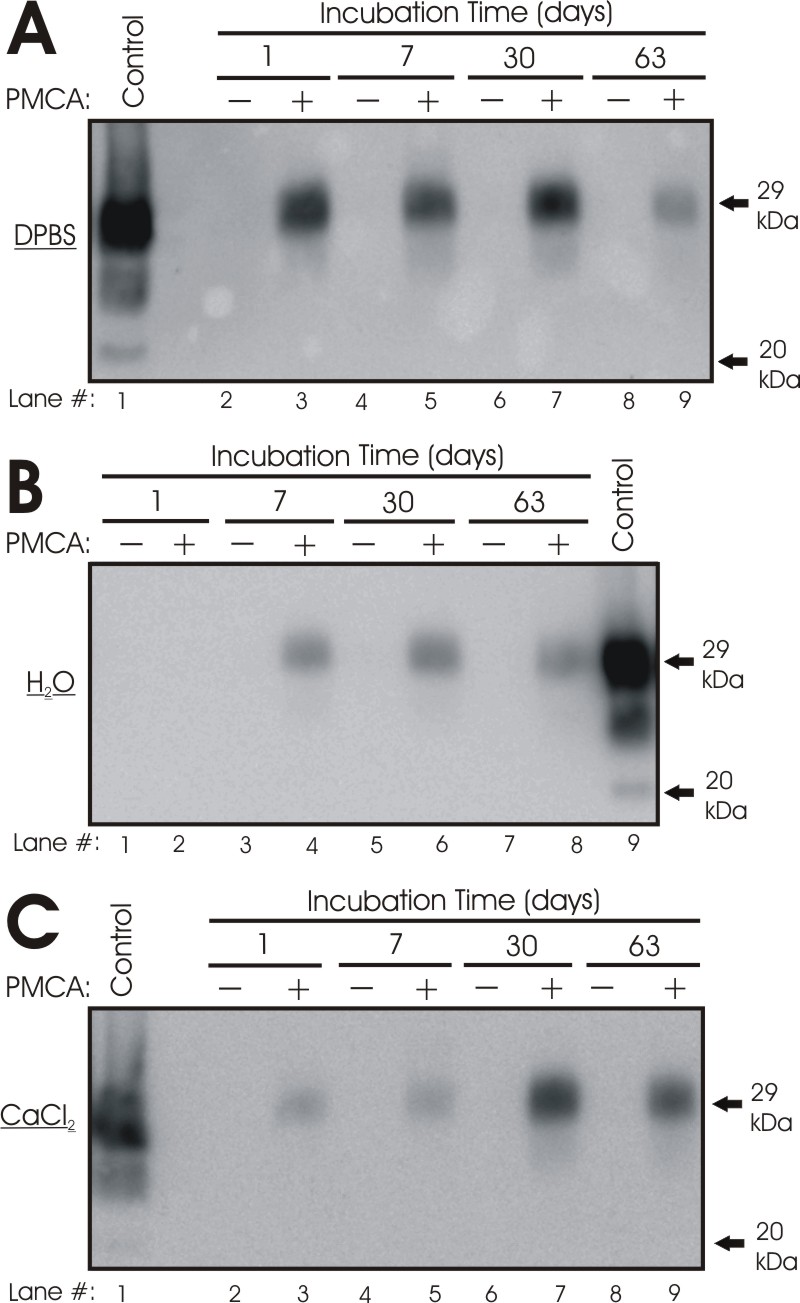


**Figure S3**. Representative immunoblots of sand-bound PMCA. **(A-C):** Representative blots of HY fine quartz sand samples subjected or not subjected to PMCA, shown with a 2 µl 10% BH control. All samples PK-digested and blotted with mAb 3F4. **(A):** HY adsorbed to fine quartz sand in DPBS with adsorption incubation time as noted. **(B):** Pure water used as the adsorption solution. **(C):** Calcium chloride used as the adsorption solution. Quantification shown in Figure 3.
