# Supplementary material for: Effects of Solution Chemistry and Aging Time on Prion Protein Adsorption and Replication of Soil-Bound Prions
Source: PLoS One. 2011 Apr 19;6(4):e18752. doi: 10.1371/journal.pone.0018752 (PMC3079715; doi:10.1371/journal.pone.0018752)
Supplement: Figure S4 — Representative immunoblots of aged soil-bound PMCA. (DOC) [file pone.0018752.s004.doc]

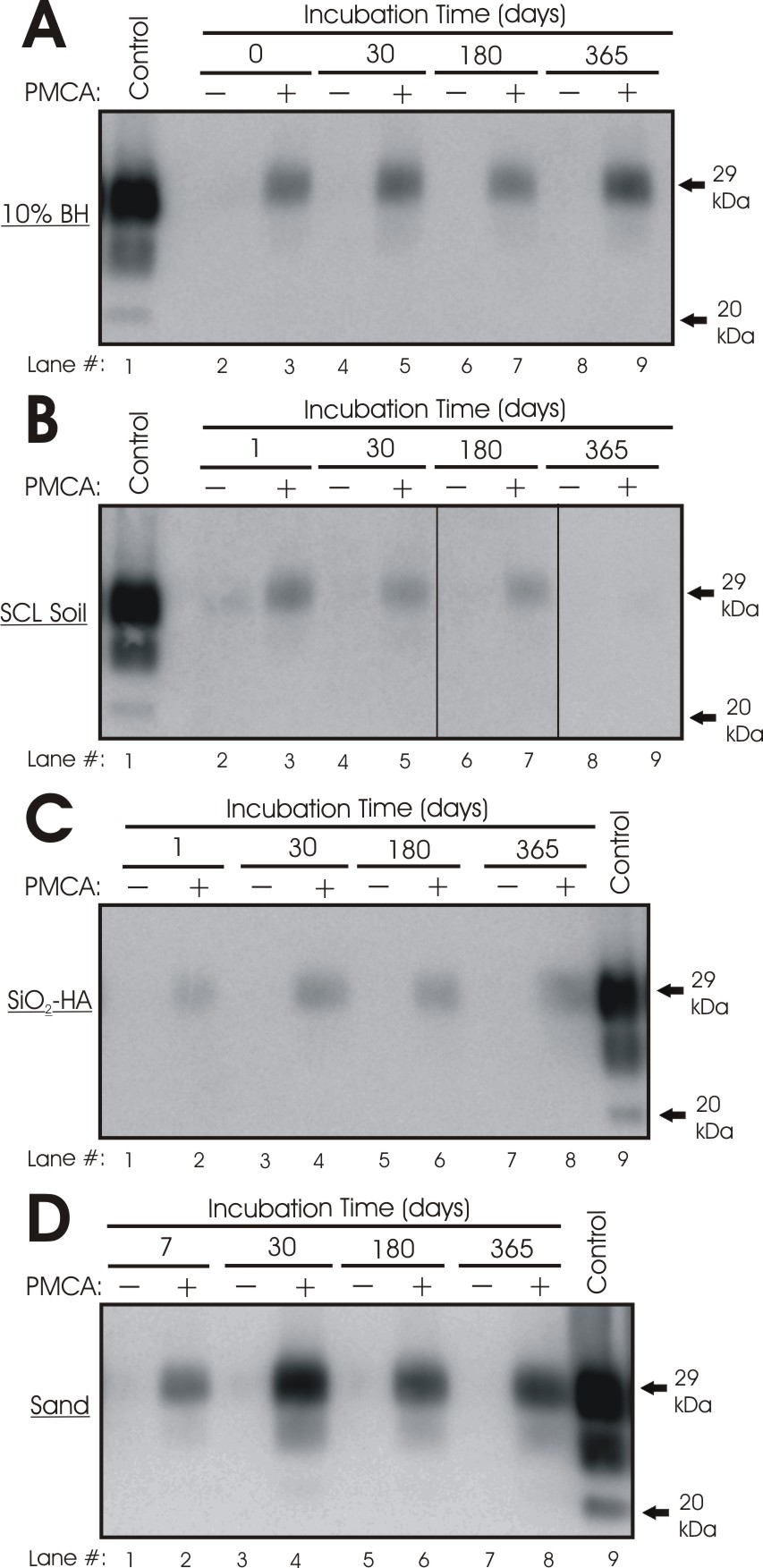


**Figure S4.** Representative immunoblots of aged soil-bound PMCA. **(A-D):** Representative blots of aged DPBS HY samples (4 µl) subjected or not subjected to PMCA, shown with a 2 µl 10% BH control. All samples PK-digested and blotted with mAb 3F4. **(A):** HY brain homogenate. **(B):** SCL Soil. **(C):** Silica beads coated with humic acid (SiO_2_-HA). **(D):** Fine quartz sand. Quantification shown in Figure 5.
