# Supplementary material for: Effects of Solution Chemistry and Aging Time on Prion Protein Adsorption and Replication of Soil-Bound Prions
Source: PLoS One. 2011 Apr 19;6(4):e18752. doi: 10.1371/journal.pone.0018752 (PMC3079715; doi:10.1371/journal.pone.0018752)
Supplement: Table S1 — Parameters of PrP adsorption to soil and soil minerals. (DOC) [file pone.0018752.s005.doc]

**Table S1.** Parameters of PrP adsorption to soil and soil minerals

| **Soil/Mineral** | **Figure Number** | **Adsorption Incubation Time** | **Brain Homogenate Treatment**^1^ | **Soil Concentration** | **Brain Homogenate** | **Amount Imaged** | **PMCA Spike**  **(1:100)** |
| --- | --- | --- | --- | --- | --- | --- | --- |
|  |  | days |  | mg soil /ml buffer | % | mg | mg |
| Bentonite Clay | 1 | 1 h, 1 d, or 7 d | Clarified^2^ | 2.5 | 0.50 | 0.25 | NA |
| “ | 2 | 1 | None | 5 | 0.50 | NA | 0.1 |
| “ | 4 | 1 | None | 2.5 | 0.50 | 0.5 | NA |
| SiO_2_ Powder | 1 | 1 h, 1 d, or 7 d | Clarified | 50 | 0.50 | 2.5 | NA |
| “ | 2 | 1 | None | 50 | 0.25 | NA | 2.0 |
| “ | 4 | 1 | None | 50 | 0.50 | 2.5 | NA |
| Fine Quartz Sand | 3 | 1 h, 1 d, or 7 d | PK-digested^3^ | 50 | 0.75 | NA | 10 |
| “ | 5 | 30 | None | 50 | 0.50 | NA | 10 |
| Rinda Silty Clay Loam | 4, 5 | 1 | None | 5 | 0.50 | 0.5 | 0.1 |
| SiO_2_-Humic Acid | 5 | 14 | None | 10 | 0.50 | NA | 1.0 |

^1^ For NaCl, H_2_O, and CaCl_2_ samples, brains homogenized in deionized water were used. For DPBS samples, brains were homogenized in DPBS.

^2^ Centrifuged for 5 min at 100 x g.

^3^ Proteinase-K digestion for 30 min at 37°C under constant agitation, stopped by 100 µg/µl pefabloc.
